# Enhanced therapeutic efficacy of doxorubicin/cyclophosphamide in combination with pitavastatin or simvastatin against breast cancer cells

**DOI:** 10.1007/s12032-023-02248-7

**Published:** 2023-12-05

**Authors:** Samar A. Dewidar, Omar Hamdy, Moetaza M. Soliman, Amal M. El Gayar, Mohamed El-Mesery

**Affiliations:** 1 Clinical Pharmacy and Pharmacy Practice Department, Faculty of Pharmacy, Mansoura University, Mansoura, Egypt; 2 Surgical oncology department, Oncology Center, Mansoura University, Mansoura, Egypt; 3 Biochemistry Department, Faculty of Pharmacy, Mansoura University, Mansoura, Egypt

**Keywords:** Neoadjuvant, Breast cancer, Statins, Chemotherapy, Tumor response

## Abstract

Fighting breast tumors mandates finding different agents devoid of chemotherapy side effects. Repurposing existing drugs, such as statins, presents a promising avenue for the development of novel cancer therapeutics. Based on the different effects of statin members, this study aims to evaluate the effect of two of the most promising lipophilic statins, Simvastatin and Pitavastatin, and their combination with a conventional chemotherapeutic regimen of doxorubicin and cyclophosphamide on breast cancer cells. MDA-MB-231 and MCF7 cell lines were used to analyze the effects of Pitavastatin and simvastatin in combination with doxorubicin/cyclophosphamide. Cell viability and cell cycle were analyzed and certain apoptosis-related genes such as Bax, Bcl2, and caspase-3, besides cyclin D1 were analyzed using qPCR. The viability of breast cancer cells decreased significantly after treatment with a doxorubicin/cyclophosphamide combination in the presence of Pitavastatin or simvastatin compared with dual doxorubicin/cyclophosphamide with a higher effect in MDA-MB-231 cells than MCF7. In MDA-MB-231, The triple combination of Pitavastatin or simvastatin with doxorubicin/cyclophosphamide resulted in an increase in the expression levels of apoptotic markers than treatment with doxorubicin/cyclophosphamide combination (Bax (p-value = 0.09& 0.02, respectively), Bax/Bcl2 ratio (*p*-value = 0.0002& <0.0001, respectively)). However, the increase in caspase3 wasn’t significant (*p*-value = 0.45& 0.09, respectively). Moreover, the expression of cyclin D1 decreased (*p*-value = 0.0002& <0.0001, respectively) and the cell cycle was arrested in the G1 phase. Combination of Pitavastatin or simvastatin with doxorubicin/ cyclophosphamide may induce apoptosis in breast cancer cells via upregulation of the Bax/Bcl2 pathway, potentially providing a promising new therapeutic strategy for breast cancer.

## Background

Breast cancer has attracted great scientific attention because it is the most common cancer type and the second-largest cause of death in the United States [1]. Breast cancer incidence has grown by 0.5% annually [1]. This alarming increase emphasizes the need to identify the underlying processes of cancer formation and develop efficient treatments [2]. Breast cell malignancy evolved from disturbance of cell signaling and uncontrolled growth factors through cascades of abnormal genetic events [3]. Molecular subtypes of breast cancer can be classified into luminal A, luminal B, Human epidermal growth receptor-2 (HER2)-enriched, and triple-negative subtypes based on the levels of mRNA gene expression [4]. Treatment options vary for breast cancer patients ranging from surgical removal of breast mass, pharmacotherapy, or using radiotherapy to kill the cancer cells. Patients with HER2-enriched and triple-negative tumors have the worst prognosis, and they should precede surgery with neoadjuvant chemotherapy regimens [4, 5].

Commonly, a regimen of double or triple chemotherapeutic drugs is initiated as soon as the diagnosis is verified [4]. For most advised regimens, doxorubicin and cyclophosphamide are used to treat breast cancer [6–10]. Despite the adequate response, these chemotherapeutic agents result in different side effects that impair the patient’s tolerability to the treatment. Identifying novel therapeutic additives is necessary to reduce the chemotherapy dose and the potential side effects.

Drug repositioning aims to discover new efficacies and direct well-known drugs for other diseases based on the pharmacological aspect of the drug to escape the conundrum of drug discovery economics and safety issues [10–12]. An example of drug repurposing is using statins in breast cancer. Cholesterol is usually essential for cell regulatory functions, conserving membrane integrity, and interacting with the extracellular matrix [13, 14]. Feedback mechanisms could closely control cholesterol levels depending on the amount of cholesterol in the cells. Unfortunately, these feedback mechanisms are impaired in cancer cells with high proliferation rates leading to the accumulation of intracellular cholesterol and activation of the HMG-CoA-enzyme [15]. Statins significantly impact cancer cells in addition to their hypocholesterolemic action, primary and secondary prevention of cardiovascular illnesses [16, 17]. Statins can inhibit tumor necrosis factor-alpha (TNF a), inhibiting angiogenesis [18]. Additionally, statins can prevent the expression of matrix metalloproteinases 2 and 9 (MMP2 & MMP9) and lower the likelihood of metastasis [19]. Moreover, statins have a suppressive effect on the cell cycle and cause G1 phase arrest, interfering with cell proliferation and migration activity [20]. It was also discovered that statins can oblige cancerous cells to evade the Warburg effect and continue through oxidative phosphorylation and activation of the tricarboxylic acid cycle (TCA) [21].

Statin members have varying degrees of physicochemical properties, including their solubility. They are divided into hydrophilic statins with hepatic availability and lipophilic statins that exert higher extrahepatic concentrations, making lipophilic members better candidates for anti-cancer agents [22]. Pitavastatin and simvastatin are members of statins that have displayed the highest anticancer activity [23]. A recent study indicated that the concomitant use of Pitavastatin with standard neoadjuvant chemotherapy protocols may improve neoadjuvant chemotherapy responses in patients with breast cancer [24]. However, further studies are required to compare the effect of different statins and to explore the molecular mechanism of action.

The current study aimed to examine whether Pitavastatin or simvastatin can enhance the anti-cancer activity of the doxorubicin/cyclophosphamide combination in breast cancer cell lines and to explore their prospective molecular mechanism of action.

## Methods

### Drugs and chemicals

In this study, doxorubicin 50 mg/25 ml vial (Dox, Adriamycin, Hikma specialized, USA) and cyclophosphamide (Cyclo, Endoxan 1gm IV vial, Baxter Oncology, USA) were used. Simvastatin and Pitavastatin were generous grants from EVA Pharma, Egypt. Other used materials include Dulbecco’s modified eagle’s medium high glucose enriched medium (DMEM, Lonza, Verviers, Belgium), fetal bovine serum (FBS, Sera laboratories international, Ltd., Brazil EU grade), phosphate buffer saline (PBS, Lonza, Verviers, Belgium) streptomycin and penicillin (Lonza, Verviers, Belgium), favor-PrepTM blood/cultured cell total RNA purification mini kit (Favorgen Biotech Corp., Ping-Tung, Taiwan), Revert Aid First Strand cDNA Synthesis Kit (Thermo Scientific, Waltham, MA, USA), HERAPLUS SYBR® Green qPCR Kit (Willowfort, Nottingham, UK) and propidium iodide (PI, ab14083, Abcam).

### Experimental cell lines

Breast cancer cell lines (M.D. Anderson - Metastatic Breast 231 (MDA-MB-231) & Michigan Cancer Foundation-7 (MCF7)) were purchased from Nawah Scientific (Almokattam, Cairo, Egypt) and grown in DMEM medium enforced with 10% FBS and 1% streptomycin/penicillin incubated under standard conditions (37^*o*^C humidified air and 5% CO2 pressure).

### Ethical approval

The ethical committee of the Faculty of Pharmacy, Mansoura University (Ref. No. 2020 − 176) approved this study.

### Cell viability analysis

MDA-MB-231 and MCF7 cells were seeded in 96-well plates with 20,000 cells/well under standard conditions. The plates were incubated to allow cell growth for 24 h before stimulation. The next day, cells were stimulated with doxorubicin (50, 25, 12.5, 6.25, 3.125 µM), cyclophosphamide (100, 50, 25, 12.5, 6.25 µM), Pitavastatin (200, 100, 50, 25, 12.5 µM), simvastatin (200, 100, 50, 25, 12.5 µM) in triplicates. Drugs were used with doxorubicin combination at concentrations around or below the resultant *IC*_50_; cyclophosphamide 100 µM, Pitavastatin 50 µM, or simvastatin 25 µM. The percentage of viable cells was detected using the crystal-violet assay technique 24 h after stimulation using a microplate reader (Bio Tek ELx800, USA) at a wavelength of 570 nm. The results are expressed as the percent of viable cells compared with the living control group (100% viability); cells grown with standard media without added drugs, negative control group (0%viablility); cells treated with a mixture of toxic compounds containing doxorubicin, dimethyl sulfoxide, sodium azide.

### Quantitative real-time PCR (qPCR)

Cells were cultivated in 6 well plates with 1 × 10^6^ cells/well in triplicates, then incubating the cells in standard conditions for 24 h before stimulation. Cells were treated with doxorubicin (10 µg/ml), cyclophosphamide (100 µM), Pitavastatin (50 µM), simvastatin (25 µM), or their combinations for gene expression. After stimulation for 24 h, cells were washed twice using cold PBS, scraped from the flask, transferred into Eppendorf tubes, centrifuged to get the precipitated cells, and discarded the supernatant. Total ribonucleic acid (RNA) was extracted using the Favor-PrepTM Blood/Cultured cell total RNA purification mini kit (Favorgen Biotech Corp., Ping-Tung, Taiwan). The first-strand cDNA was formed using the Revert Aid First Strand cDNA Synthesis Kit (Thermo Scientific, Waltham, MA, USA). HERAPLUS SYBR® Green qPCR Kit (Willowfort, Nottingham, UK) was used in (qPCR) following the manufacturer protocol. Using the 2^−∆∆ct^ method, gene expression fold changes were determined and presented as an average of three independent experiments (Livak and Schmittgen, 2001). Table 1. shows the primer sequences used in qPCR for caspase-3, Bax, BCL-2, and cyclin D1.

**Table 1:** Primer sequences used in qPCR

	Forward (5’-3’)	Reverse (5’-3’)
Caspase-3	5’-ACATGGAAGCGAATCAATGGACTC-3’	5’-AAGGACTCAAATTCTGTTGCCACC-3’
Cyclin D1	5’-AGACCTGCGCGCCCTCGGTG-3’	5’-GTAGTAGGACAGGAAGTTGTTC-3’
Bax	5’-CCCGAGAGGTCTTTTTCCGAG-3’	5’-CCAGCCCATGATGGTTCTGAT-3’
BCL-2	5’-TGTGGCCTTCTTTGAGTTCGGTG-3’	5’-GGTGCCGGTTCAGGTACTCAGTCA-3’

### Cell cycle analysis

Cells were seeded into 6 well plates at a density of 1 × 10^6^ well, maintained in standard conditions for 24 h to allow their adhesion. Following centrifugation at 1800 rpm and removal of the supernatant, cells were permeabilized and fixed in 1 ml of cold 96% or absolute ethanol in ice, which was added dropwise, while vortexing to ensure the fixation of all cells with minimum clumping. The tubes stood for 15 min before centrifugation for 10 min at 1800, followed by aspiration of alcohol without disturbing the pellets [25]. Then, pellets were resuspended in 1 ml of propidium iodide (PI) buffer (25 µg/mL PI, 500 mg sodium citrate, and 0.5 ml Triton X-100 to 500 ml distilled water). Cells were suspended in 1 ml of the staining solution at 4 °C for 30 min and maintained in ice. Afterward, they were filtered through 30 μm nylon mesh to remove nuclear aggregates in another 5 ml tube. DNA content was measured using Accuri™ C6, and G0/G1, S, and G2/M cells were appropriately gated using Accuri™ C6 Software.

### Statistical analysis

Data were expressed as mean ± standard error of the mean (SEM). One-way ANOVA followed by Tukey-Kramer, multiple comparison test, was performed using GraphPad Prism version 9.0.0 for Windows; “GraphPad Software, San Diego, California USA, www.graphpad.com”. The significance level was at a *P*-value of ˂0.05.

## Results

### Pitavastatin and simvastatin decrease cell viability of MDA-MB-231 and MCF7 cell line

**Fig. 1:**
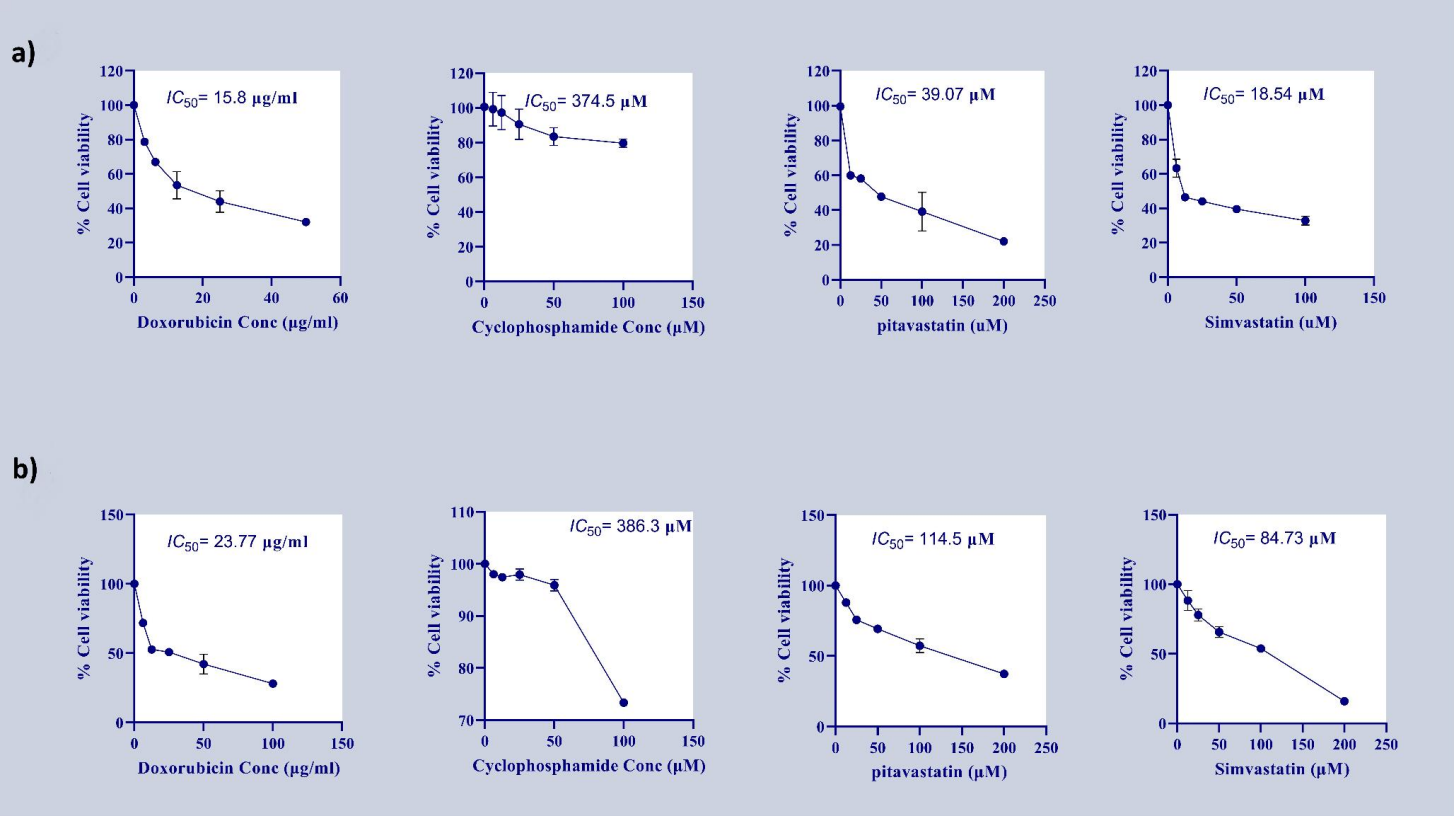
The viability of MDA-MB-231 **(A)** and MCF7 **(B)** cells treated with different concentration gradient of doxorubicin (Dox), cyclophosphamide (Cyclo), Pitavastatin and simvastatin to determine inhibitory concentration 50 for 24 h. Cells were treated with the indicated concentrations for 24 h. Then, the cell viability was analyzed using crystal violet staining

MDA-MB-231 and MCF7 cell lines were treated with different concentrations of chemotherapies (doxorubicin (Dox) and cyclophosphamide (Cyclo)) and statin drugs (Pitavastatin and simvastatin) to determine (*IC*_50_) for each agent. As illustrated in Fig. 1, MCF7 required higher concentrations to kill 50% of cells than MDA-MB-231.

**Fig. 2:**
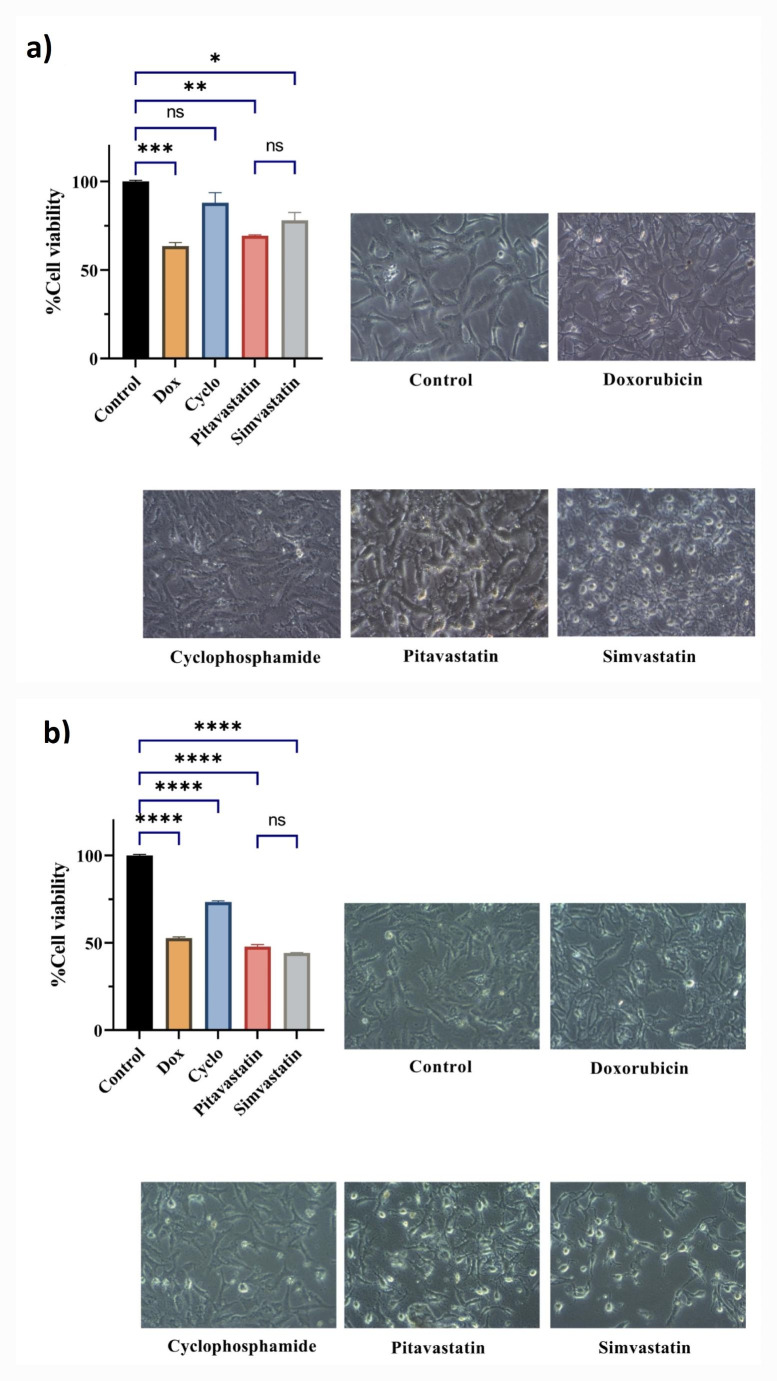
The change in cell viability percent and their microscopic pictures using (100x power) on treatment with each drug alone in human breast cancer cell lines; MDA-MB-231 **(A)** and MCF7 **(B)**. Control sets as untreated cells; Dox: doxorubicin (6.25 µg/ml) in MDA-MB-231 and (12.5 µg/ml) in MCF7, Cyclo: cyclophosphamide (100 µM), Pitavastatin (50 µM), simvastatin (25 µM). The values are considered statistically significant compared to solvent-treated control at * *p* < 0.05, ** *p* < 0.01, *** *p* < 0.001, **** *P* < 0.0001, and ns means not significant

In MDA-MB-231 cells (Fig. 2a), treatment with every drug alone decreased cell viability relative to the untreated control significantly except cyclophosphamide which decreased the viable cells only to 87.94%. Treatment with either Pitavastatin or simvastatin comparably decreased the percent cell viability to 69.2% and 77.9% respectively (*P*-value = 0.557). Treatment with doxorubicin decreased cell viability to 63.43% but did not show superiority over either Pitavastatin or simvastatin (*P*-value = 0.8256 and 0.0963 respectively).

Whereas in MCF7 cells (Fig. 2), treatment with all agents significantly decreased the percent of viable cells as compared with the untreated cells. Pitavastatin decreased the cell viability to 47.75% instead of 52.70% in the doxorubicin group (*P*-value = 0.0208). Simvastatin decreased the viability of cells to 44.2% which is significantly lower than doxorubicin (*P*-value = 0.0013) and induced a similar level of cell death as Pitavastatin (*P*-value = 0.085).

**Fig. 3:**
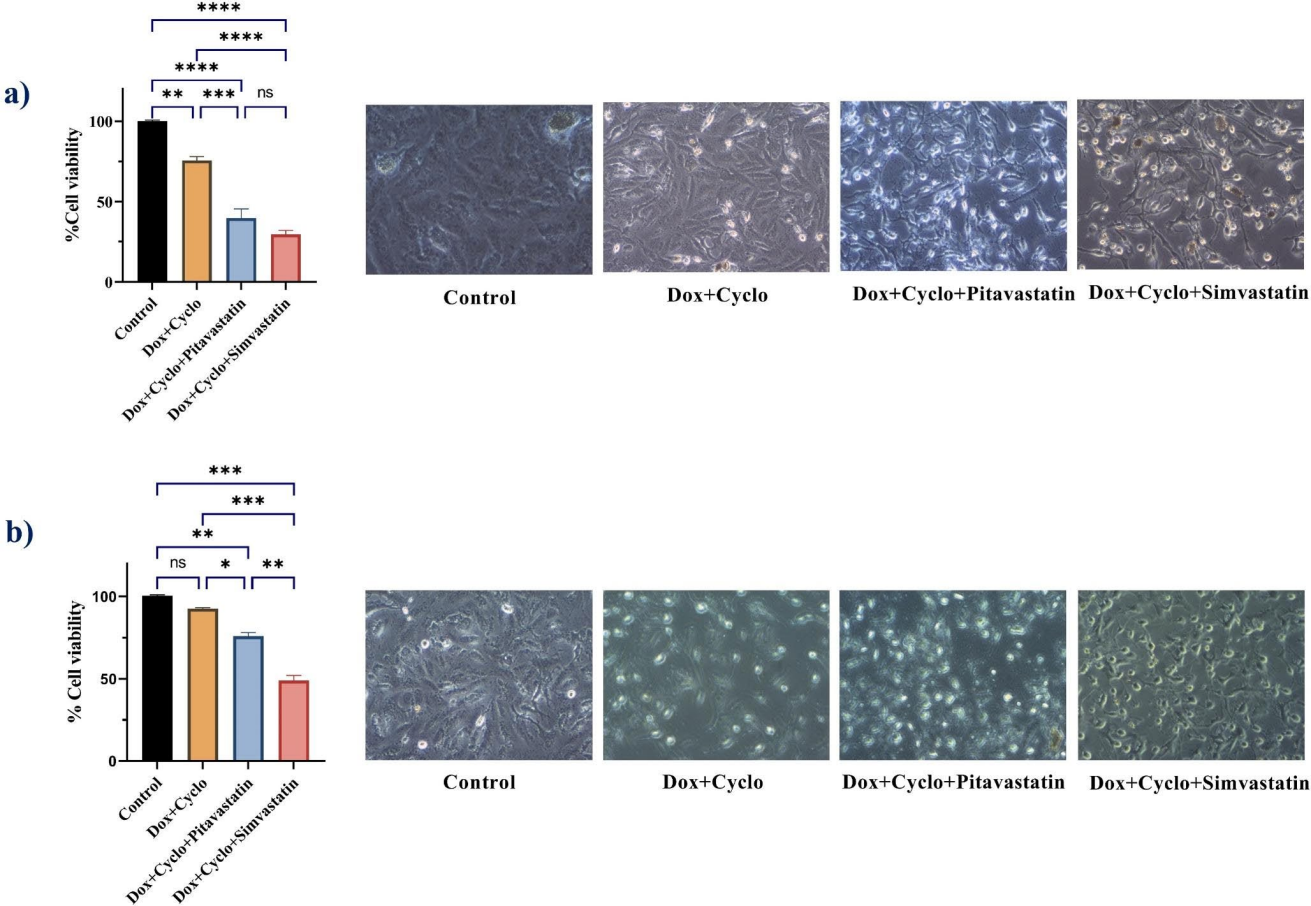
The change in cell viability percent and their microscopic pictures (100x power) on treatment with drug combinations in human breast cancer cell lines; MDA-MB-231 **(A)** and MCF7 **(B)**. Control sets as untreated cells; Dox: doxorubicin (6.25 µg/ml) in MDA-MB-231 and (12.5 µg/ml) in MCF7, Cyclo: cyclophosphamide (100 µM), Pitavastatin (50 µM), simvastatin (25 µM). The values are considered statistically significant compared to solvent-treated control at * *p* < 0.05, ** *p* < 0.01, *** *p* < 0.001, **** *P* < 0.0001, and ns means not significant

Figure 3 demonstrates that treatment with doxorubicin/cyclophosphamide decreased the cell viability to 75.48% in MDA-MB-231 but only to 92.58% in MCF7.

Pretreatment with Pitavastatin followed by stimulating the cells with doxorubicin/ cyclophosphamide combination decreased the viability of MDA-MB-231 cells to 39.65% (Fig. 3a) and 75.82% in MCF7 cells (Fig. 3b). Similarly, pretreatment of MDA-MB-231 cells with simvastatin followed by doxorubicin/cyclophosphamide combination lowered the percentage of viable cells to 29.51% and 48.94% in MCF7 cells (Fig. 3). According to our results, the simvastatin triple combination resulted in a much more cytotoxic effect than the Pitavastatin triple combination in MCF7 cells (*P*-value = 0.002) while their effect on MDA-MB-231 cells was similar (*P*-value = 0.223) (Fig. 3**).**

### Pitavastatin and simvastatin arrest G0/G1 phase in MDA-MB-231 and MCF7 cell lines

**Fig. 4:**
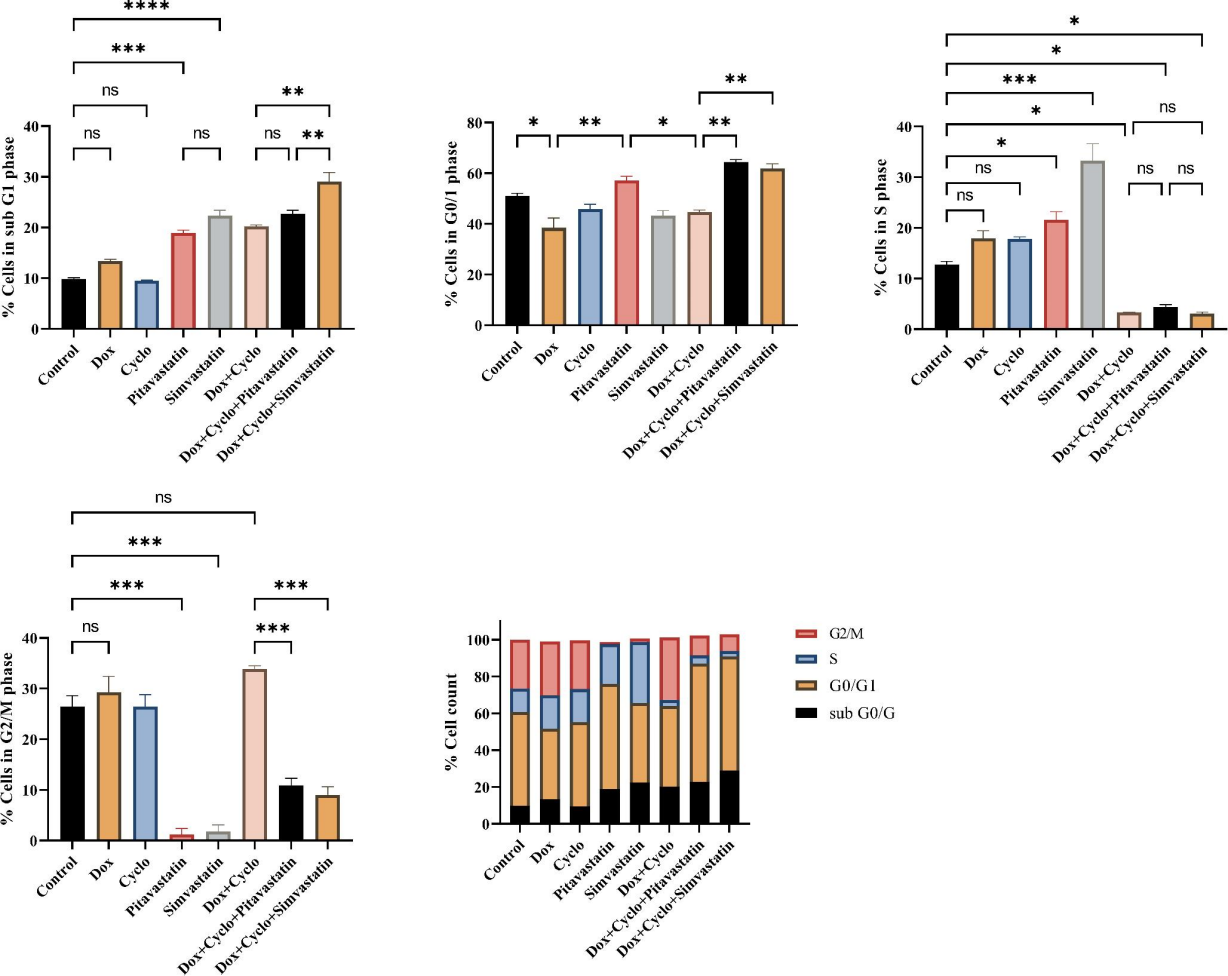
The change in percentage of human breast cancer cells MDA-MB-231 in different cell cycle phases using flow cytometry **(E)**, percentage in sub-G0/G1 phase **(A)**, Percentage in G0/G1 phase **(B)**, Percentage in S phase **(C)** and Percentage in G2/M phase **(D)**. Control sets as untreated cells; Dox: doxorubicin (6.25 µg/ml), Cyclo: cyclophosphamide (100 µM), Pitavastatin (50 µM), simvastatin (25 µM). The values are considered statistically significant compared to solvent-treated control at * *p* < 0.05, ** *p* < 0.01, *** *p* < 0.001, **** *p* < 0.0001, and ns means not significant

**Fig. 5:**
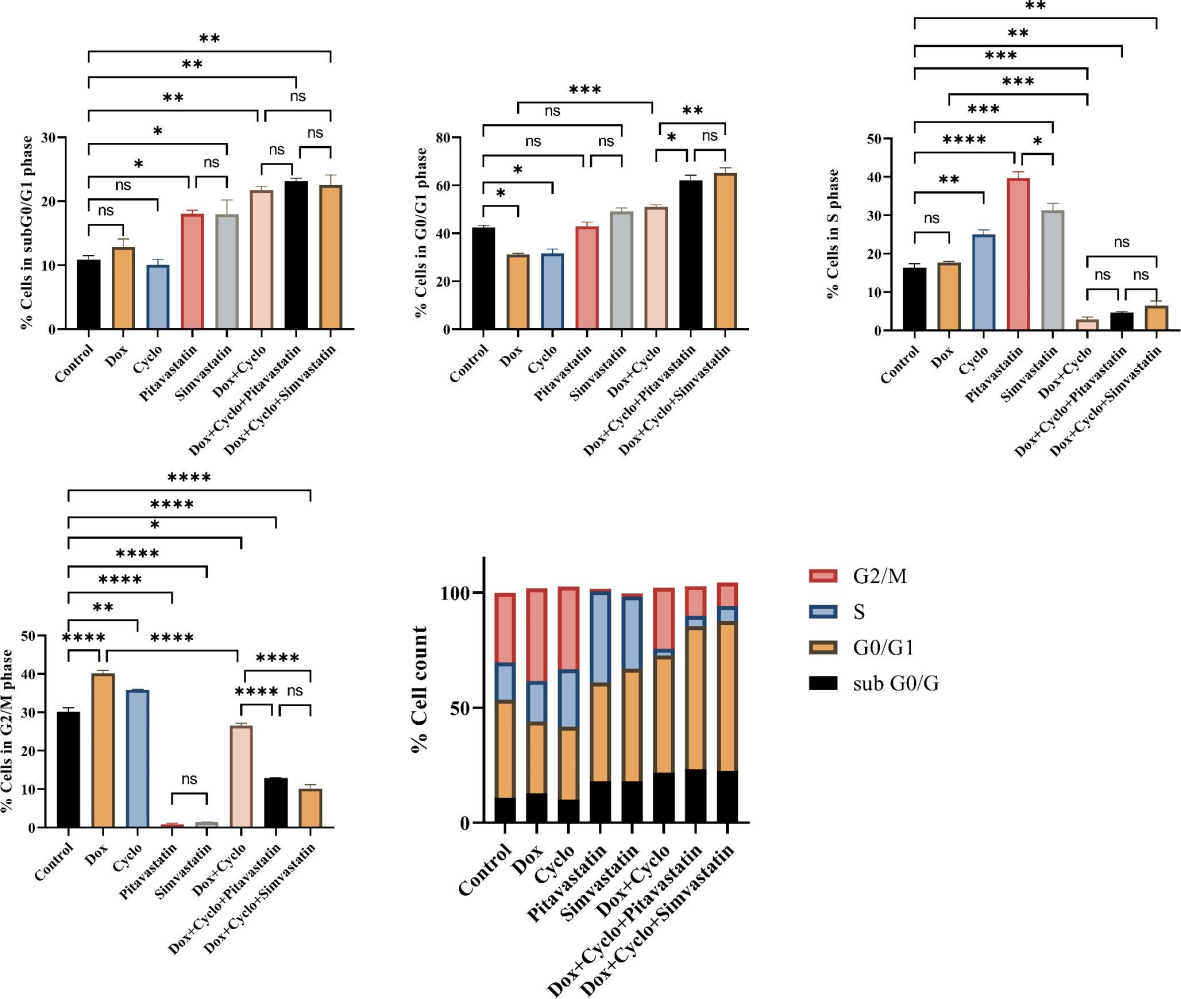
The change in percentage of human breast cancer cells MCF7 in different cell cycle phases using flow cytometry **(E)**, percentage in sub-G0/G1 phase **(A)**, Percentage in G0/G1 phase **(B)**, Percentage in S phase **(C)** and Percentage in G2/M phase **(D)**. Control sets as untreated cells; Dox: doxorubicin (6.25 µg/ml), Cyclo: cyclophosphamide (100 µM), Pitavastatin (50 µM), simvastatin (25 µM). The values are considered statistically significant compared to solvent-treated control at * *p* < 0.05, ** *p* < 0.01, *** *p* < 0.001, **** *p* < 0.0001, and ns means not significant

After 24 h of incubation, DNA content was assessed in cells using PI staining flow cytometry. Percentages of cells in each cell phase (G0/G1, S, and G2/M) are presented in Tables 2 and 3. We observed that statin members (Pitavastatin and simvastatin) and all combination treatments arrested cell cycle progression at the G0/G1 phase in MDA-MB-231 & MCF7 cells. The percentages of the sub-G1 populations of both cells were maximally increased in statin combination with the doxorubicin/ cyclophosphamide treated group than in the untreated control group (Fig. 4 and Fig. 5**).** Statin members had a similar effect on cell cycle distribution in both cell lines. As shown in Figs. 4 and 5, the cell populations in G0/G1 phase increased from 50.95% in control cells to 64.35% (Pitavastatin triple combination) and 61.85% (simvastatin triple combination) treated cells in MDA-MB-231 cells and from 42.5% in control cells to 62.15% ((Pitavastatin triple combination) and 65.2% (simvastatin triple combination) in MCF7 cells.

**Table 2:** Cell cycle analysis of MDA-MB-231 cells after treatment for 24 h showing the DNA content (mean ± SEM) at different cycle phases

Drug	Cell cycle phases			
	SubG0/G1	G0/1	S	G2/M
Control	9.8 ± 0.30	50.95 ± 1.15	12.70 ± 0.70	26.45 ± 2.15
Dox	13.35 ± 0.35	38.40 ± 3.90	17.95 ± 1.45	29.3 ± 3.10
Cyclo	9.50 ± 0.10	45.85 ± 1.95	17.85 ± 0.35	26.45 ± 2.35
Pitavastatin	18.90 ± 0.60	57.10 ± 1.7	21.55 ± 1.65	1.25 ± 1.15
Simvastatin	22.35 ± 1.05	43.25 ± 2.05	33.20 ± 3.40	1.85 ± 1.25
Dox/ cyclo	20.20 ± 0.30	44.75 ± 0.75	3.35 ± 0.05	33.85 ± 0.65
Dox/ cyclo + Pitavastatin	22.7 ± 0.70	64.35 ± 1.05	4.350 ± 0.45	10.90 ± 1.40
Dox/ cyclo + simvastatin	29.05 ± 1.85	61.85 ± 1.85	3.05 ± 0.35	9.05 ± 1.55

G0/G1: growth phase 0/1, S: synthesis phase, G2/M: growth phase 2 and mitosis phase. Control sets as untreated cells; Dox: doxorubicin (6.25 µg/ml) in MDA-MB-231, Cyclo: cyclophosphamide (100 µM), Pitavastatin (50 µM), simvastatin (25 µM).

**Table 3:** Cell cycle analysis of MCF7 cells after treatment for 24 h showing the DNA content (mean ± SEM) at different cycle phases

Drug	Cell cycle phases			
	SubG0/G1	G0/1	S	G2/M
Control	10.90 ± 0.60	42.5 ± 0.8	16.3 ± 1.10	30.1 ± 1.10
Dox	12.86 ± 1.25	31.1 ± 0.60	17.65 ± 0.35	40.2 ± 0.70
Cyclo	10.05 ± 0.85	31.7 ± 1.70	25 ± 1.20	35.8 ± 0.20
Pitavastatin	18.00 ± 0.60	42.9 ± 1.80	39.7 ± 1.60	0.9 ± 0.20
Simvastatin	17.95 ± 2.25	49.05 ± 1.55	31.25 ± 1.85	1.4 ± 0.10
Dox/ cyclo	21.75 ± 0.55	51 ± 0.90	2.85 ± 0.65	26.5 ± 0.70
Dox/ cyclo + Pitavastatin	23.15 ± 0.45	62.15 ± 2.05	4.6 ± 0.30	12.9 ± 0.10
Dox/ cyclo + simvastatin	22.50 ± 1.60	65.2 ± 2.10	6.5 ± 1.20	10.2 ± 10

G0/G1: growth phase 0/1, S: synthesis phase, G2/M: growth phase 2, and mitosis phase. Control sets as untreated cells; Dox: doxorubicin (12.5 µg/ml), Cyclo: cyclophosphamide (100 µM), Pitavastatin (50 µM), simvastatin (25 µM).

### Pretreatment of Breast cancer cells with either pitavastatin or simvastatin enhances apoptosis induction

**Fig. 6:**
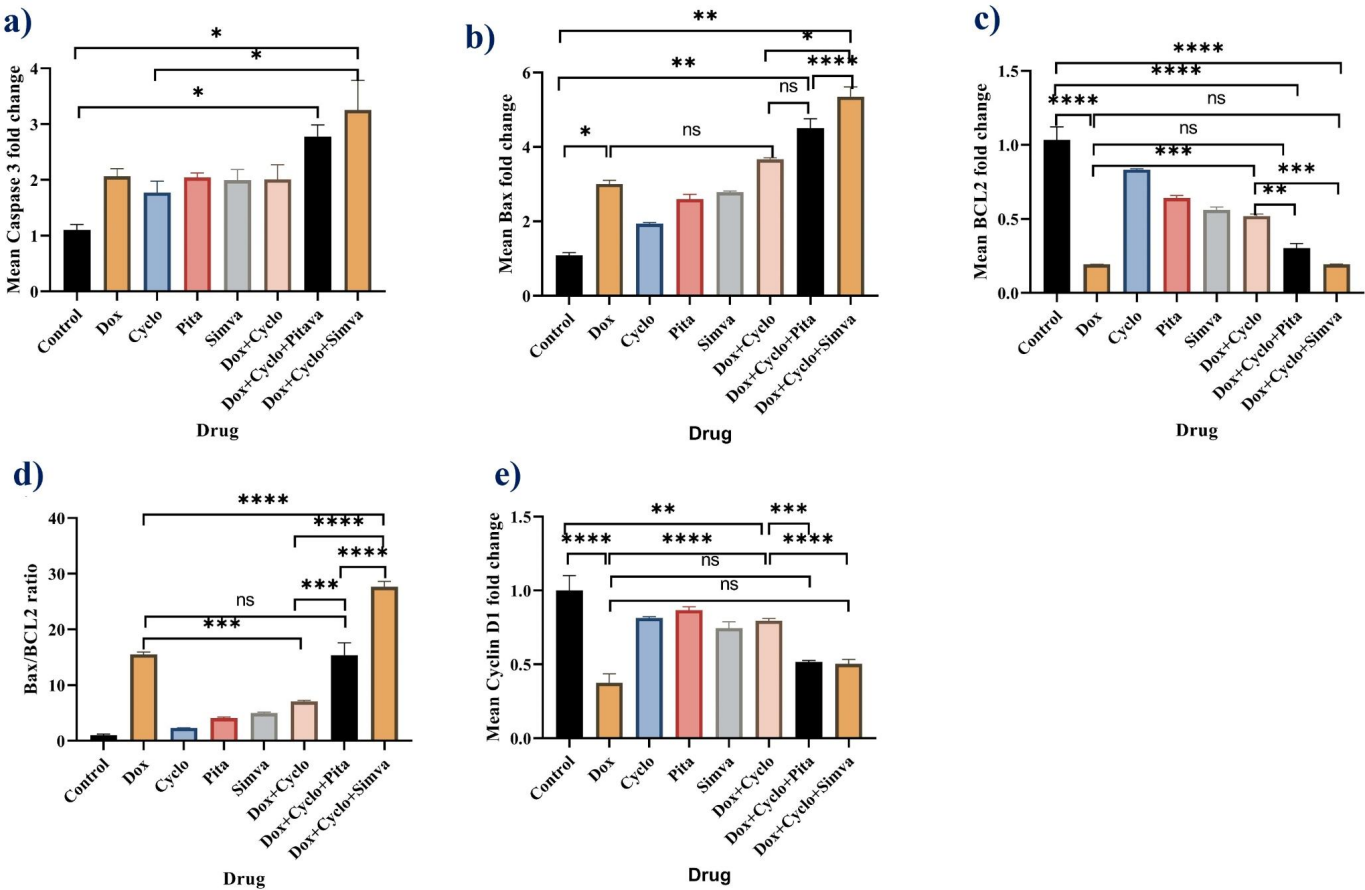
Analysis of mRNA expression of caspase 3 **(A)**, Bax **(B)**, Bcl2 **(C)**, the ratio between Bax/Bcl2 **(D)**, cyclin D1 **(E)** after treatment of MDA-MB-231 cells with each drug alone and their combinations related to control, 0.1% DMSO, Dox: doxorubicin (6.5 µg/ml), Cyclo: cyclophosphamide (100 µM), Pitavastatin (50 µM), simvastatin (25 µM). The values are considered statistically significant compared to solvent-treated control at * *p* < 0.05, ** *p* < 0.01, *** *p* < 0.001, **** *P* < 0.0001, ns means not significant

Pretreatment with Pitavastatin or simvastatin significantly increased caspase 3 gene expression in MDA-MB-231 cells following stimulation with Dox/ Cyclo (*P*-value = 0.02 and 0.004, respectively, Fig. 6a).

The B-cell lymphoma-2-associated X protein (Bax), which regulates apoptosis, increased significantly regarding its gene expression when cells were treated with Dox compared to untreated cells (*P*-value = 0.0385). Furthermore, the combination of Dox/ Cyclo resulted in a further increase in Bax levels (*P*-value < 0.0001), and this increase was even more pronounced when a statin drug such as Pitavastatin or simvastatin was added to the Dox/ Cyclo combination (*P*-values < 0.0067 and 0.0028, respectively when compared with untreated cells). (Fig. 6b)

The apoptotic suppressor Bcl2 gene that encodes B-cell lymphoma-2 expression level was decreased mainly in the Dox group compared to the untreated cells (*P*-value < 0.0001). When Dox was combined with Cyclo, the expression levels increased significantly compared to treatment with Dox alone (*P*-value = 0.0001). Adding a statin member to the Dox/ Cyclo combination resulted in decreased Bcl2 expression levels similar to the expression levels observed with Dox alone (*P*-value = 0.391 with Pitavastatin and > 0.999 with simvastatin addition, Fig. 6c).

As illustrated in Fig. 6d, the ratio between Bax/ Bcl2 increased significantly upon treatment with different agents related to the control group. However, triple treatment of either Pitavastatin or simvastatin with Dox/ Cyclo showed the maximum Bax/Bcl2 ratio at a *P*-value < 0.0001. Cells treated with the combination of Pitavastatin with Dox/ Cyclo did not significantly change from doxorubicin alone (*P*-value > 0.99) while in simvastatin combination the increase was significant (*P*-value < 0.0001).

MDA-MB-231 cells treated with Dox minimally expressed the proliferative cyclin D1. Nevertheless, the treatment combination of Dox/ Cyclo increased cyclin D1 expression more than treatment with Dox as a single agent (*P*-value = 0.002). Upon addition of Pitavastatin or simvastatin to Dox/ Cyclo combination, the expression of cyclin D1 decreased significantly in comparison with Dox/ Cyclo (*P*-value = 0.0002 and < 0.0001, respectively, Fig. 6e).

## Discussion

Doxorubicin is frequently used as a chemotherapeutic agent in different regimens for treating breast cancer in combination with cyclophosphamide in each cycle [6, 10, 26]. Comprehensive strategies with minimal adverse effects and maximum therapeutic response can be evolved from combination strategies of different therapeutic agents at lower doses to result in better response and decreased drug resistance [27]. In addition, drug repurposing sheds light on statins as anti-cancer agents, and using them in combination with conventional chemotherapy would offer many benefits for the patients [28–30].

This study evaluated the effect of two lipophilic statin members (simvastatin and Pitavastatin) with the most popular chemotherapy regimen of doxorubicin/ cyclophosphamide in ER-positive breast cancer cells (MCF7) and triple-negative breast cancer cells (MDA-MB-231).

According to our results, the *IC50* of analyzed drugs was higher in the MCF7 cell line than in MDA-MB-231, indicating that MCF7 may be more resistant than MDA-MB-231 cells against the analyzed drugs in this study. A previous study mentioned that the positivity of hormonal receptor expression lowers the chemotherapy treatment response [31]. This was in concordance with another study on different cell lines and had revealed that *IC*_50_ was lower for triple-negative cell lines, represented here by MDA-MB-231 cells than non-triple negative cells (MCF7) [32, 33]. Consequently, the MDA-MB-231 cell line showed lower viability than MCF7 after treatment with combination therapies. Treatment of cells with doxorubicin/ cyclophosphamide with Pitavastatin or simvastatin decreased the cellular resistance to chemotherapy resulting in the lowest cell viability percentage on treatment, especially for MDA-MB-231 cells. However, this contradicts Rezano et al. findings, which stated that simvastatin’s synergistic activity with doxorubicin was produced in MCF7 but not MDA-MB-231 cells [33]. The difference may be attributed to the presence of cyclophosphamide in the treatment combination in our study. The findings of this study mean that combination treatment with statin members would offer more cell death in MDA-MB-231 cells than MCF7. The observed difference in the response of MCF7 and MDA-MB-231 cells to statins may be due to the variation in receptor expression status between the two cell types. Specifically, MCF7 cells express estrogen receptor (ER), whereas MDA-MB-231 cells do not. This confirmed that statins can decrease cell proliferation and progression mainly on ER-negative breast cancer subtypes [23, 34]. Furthermore, MDA-MB-231 cells harbor a mutation in the p53 gene and exhibit overexpression of the mevalonate pathway, rendering them more susceptible to the effects of statins [32]. Moreover, MDA-MB-231 cells express pituitary tumor transforming gene 1 (PTTG1), which is markedly suppressed using statins, leading to diminished cell invasion due to decreased matrix metalloproteinase-2 (MMP2) and matrix metalloproteinase-9 (MMP9) activity [35, 36].

Both Pitavastatin and simvastatin produced antiproliferative activity, evidenced by the decreased expression level of cyclin D1 than the untreated cells. Cyclin D1 is implicated in regulating cell division and G1/S transition [37], and its overexpression is related to malignant transition [38]. The current study found that simvastatin and Pitavastatin in combination with doxorubicin/ cyclophosphamide significantly reduced cyclin D1 expression levels more than combined doxorubicin/ cyclophosphamide in the MDA-MB-231 cell line. In this context, the flow cytometric analysis of the cell cycle demonstrated that the cell-cycle progression of MDA-MB-231 cells was arrested in the G1 phase and accumulated in the G0/1 phase, and the arrest was significant in the triple combination-treated group rather than in other treated groups. These findings demonstrate that simvastatin and Pitavastatin inhibit MDA-MB-231 cell proliferation by inducing cell-cycle arrest. This was compatible with a study that stated that statin could upregulate cyclin-dependent kinase inhibitors causing G1/S arrest [39].

Apoptosis, as a naturally orchestrated mechanism, occurs physiologically with a pivotal role in tumor preventive effect and also can participate in the chemotherapeutic response by playing an important target for treatment strategies resulting in the activation of different pathways inhibiting the malignant transformation of different cells and hence preventing resistance [40]. The present study showed that Pitavastatin or simvastatin alone increases the expression of the apoptotic markers to a degree that is comparable to that induced by chemotherapeutic agents such as doxorubicin and cyclophosphamide. Furthermore, combining Pitavastatin or simvastatin with doxorubicin/ cyclophosphamide enhanced apoptotic activity in MDA-MB-231 and MCF7 cells. These findings are in agreement with the results reported by Buranrat et al. [41]. Apoptosis is evidenced by a significantly decreased expression of the antiapoptotic Bcl2 gene with increased expression of proapoptotic Bax and subsequently increase in the ratio of Bax/Bcl2. Moreover, there was a maximal increase in the activity of caspase-3 when statins were combined with doxorubicin/cyclophosphamide, as demonstrated by our prior clinical study where the addition of Pitavastatin to a doxorubicin/cyclophosphamide regimen resulted in a rise in the serum level of caspase-3 when compared to patients who received chemotherapy regimen alone [24]. The elevated apoptotic activity was higher in the simvastatin combination with doxorubicin/ cyclophosphamide than in the Pitavastatin combination. This may indicate that simvastatin would minimize the dosage of chemotherapy and hence decrease the toxicity. These findings were confirmed by cell cycle analysis, which revealed a rise in the proportion of apoptotic cell fragments in the sub-G0 phase. While the percentage of apoptotic cells did not differ significantly between doxorubicin/cyclophosphamide co-treatment and doxorubicin treatment alone, the administration of either Pitavastatin or simvastatin in combination with doxorubicin/cyclophosphamide resulted in a notable increase in apoptotic fragments. Furthermore, triple treatment with doxorubicin/ cyclophosphamide with Pitavastatin or simvastatin resulted in arresting cell progression at the G0/1 phase in both cell lines, similarly in other cancer cells such as hepatocellular carcinoma and prostate cancer [42, 43].

In agreement with our study, statins can induce apoptosis by activating the intrinsic mitochondrial pathway which involves reducing mitochondrial membrane potential and releasing the mitochondrial activator of caspases, Smac/DIABLO [44].

Additionally, they upregulate the expression of proapoptotic proteins Bax and activate procaspases 3, 7, 8, and 9, and Bim while downregulating the antiapoptotic protein Bcl2 [44–47].

## Conclusion

This study confirms the ability of Pitavastatin and simvastatin to potentiate the anti-cancer activity of the doxorubicin/ cyclophosphamide combination against MCF-7 and MDA-MB-231 breast cancer cells. The detected enhancement of the anticancer activity may be attributed to the induction of apoptosis, inhibiting proliferation, and arresting the cell cycle. In view of the results of this study, statins represent a novel combination therapy with known chemotherapeutic drugs to enhance their efficacy that may lead to a decrease in the required doses and hence minimize the adverse effects in cancer patients.

## Data Availability

All the data used in this manuscript is available on request.
